# *met1* DNA Methyltransferase Controls *TERT* Gene Expression: A New Insight to The Role of Telomerase in Development

**DOI:** 10.22074/cellj.2020.6290

**Published:** 2019-09-08

**Authors:** Maryam Zangi, Mohammad Bagher Bagherieh Najjar, Masoud Golalipour, Mahnaz ghdasi

**Affiliations:** 1 Department of Biology, Faculty of Science, Golestan University, Gorgan, Iran; 2 Cellular and Molecular Research Center, Golestan University of Medical Sciences, Gorgan, Iran

**Keywords:** Developmental Abnormalities, *met1*, Telomerase

## Abstract

**Objective:**

DNA methylation systems are essential for proper embryo development. Methylation defects lead to developmental abnormalities. Furthermore, changes in telomerase gene expression can affect stability of chromosomes and produces abnormal growth. Therefore, defects in both methylation and telomerase gene expression can lead to developmental abnormalities. We hypothesized that mutation in the methylation systems may induce developmental abnormalities through changing telomerase gene expression.

**Materials and Methods:**

In this experimental study, we used Arabidopsis thaliana (At) as a developmental model. DNA was extracted from seedlings leaves. The grown plants were screened using polymerase chain reaction (PCR) reactions. Total RNA was isolated from the mature leaves, stems and flowers of wild type and met1 mutants. For gene expression analysis, cDNA was synthesized and then quantitative reverse transcription PCR (qRT-PCR) was performed.

**Results:**

Telomerase gene expression level in homozygous *met1* mutant plants showed ~14 fold increase compared to normal plants. Furthermore, *TERT* expression in met1 heterozygous was~ 2 fold higher than the wild type plants.

**Conclusion:**

Our results suggested that *TERT* is a methyltransferase-regulated gene which may be involved in developmental abnormities causing by mutation in *met1* methyltransferase system.

## Introduction

Cytosine methylation is an important epigenetic feature which can be preserved after each round of DNA replication (1). Methylation of cytosine is found in CG, CNG (“N” any nucleotide) and CHH (any asymmetric site, H= A, C or T) sequences (2). Methylation at CG sites in mammals is maintained by DNA methyltransferase *Dnmt1* (3). Mouse *Dnmt1* and *Arabidopsis met1* are orthologues of human *DNMT1*. 

 Both MET1 and DNMT1 possess large N termini containing bromo-adjacent homology (BAH) domains (4-8). *Dnmt1* mutant mice die nine days after development start. However, *met1 Arabidopsis* mutants with several developmental abnormalities, like reduced apical dominance, altered leaf shape (curled leaves) and altered flowering time, are alive making *Arabidopsis* a suitable model for research on methylation system defects (9, 10). met1 is responsible for 80-90% of methylation on *Arabidopsis* genome (11). The number of met1 homozygous mutant individuals from heterozygous parents is only 2% of Mendel’s prediction (12). Few genes, such as *fwa* and *superman*, were found in the *met1* mutant plants to be misregulated, while they are responsible for some developmental phenotypes (13, 14). On the other hand, in mouse embryonic cells, methylation system defects lead to telomere elongation change. Defect in telomerase gene expression was also reported to cause developmental abnormalities (15). Telomere has a crucial role in chromosome stability and replication. Therefore, changes in telomerase gene expression can influence growth and development (16). Telomerase gene in plants is developmentally regulated, similar to the regulation mechanism in humans (17, 18). Expression analysis revealed that *TERT* overexpression modulate expression of some genes needed to increase longevity (19). 

Methylation defects and telomerase down-regulation both lead to developmental abnormalities. Therefore, we hypothesized that mutation in methylation system induces developmental abnormalities through changing telomerase expression. 

## Materials and Methods

### Plant material and growth condition

All *met1* heterozygous seeds in this study were kindly provided by Prof. Poszkowski laboratory, University of Geneva, Switzerland. *Arabidopsis met1^+/-^* heterozygous seeds were grown in the mixture of forest soil and moss with 1:1 ratio, or mixture of forest soil, vermiculite and perlite with 4:3:2 ratios. They were grown at growth chambers at 23°C using a 16 hours light/8 hours dark photoperiod. After growing seedlings, BASTA was sprayed on 7-10 days old seedlings and the sensitive plants were removed. Then, seedlings with serrated leaf margins were sampled for genotyping. 

### DNA and RNA extraction

DNA was extracted from leaves of seedlings, using Dellaporta et al. (20) method. After grinding in liquid nitrogen, 500 µl buffer [Tris-Hcl: 1 M, pH=9.0, LiCl: 2 M, Ethylenediaminetetraacetic acid (EDTA): 0.5 M, 10% w/v sodium dodecyl sulfate (SDS)] was added to them. After five minutes spinning at high speed, 350 µl of supernatant was transferred into a microtube containing 350 µl isopropanol and spun 10 minutes at high speed. The liquid was poured off and the pellet was dried. After that 100-200 µl ddH_2_O was added and shacked at room temperature for 30 minutes. Total RNAwas extracted from mature wild type, *met1* heterozygous and homozygous mutant plants according to Dellaporta et al. (20) method. Briefly, 0.1-0.5 g of fresh tissue was grinded in liquid nitrogen and transferred into a microtube containing 750 µl of extraction buffer (Tris-Hcl: 100 mM, pH=8.5, NaCl: 100 mM, EDTA: 20 mM, 1% Sarkosyl) and 750 µl of phenol/chloroform. Then the standard protocol of phenol/ chloroform extraction was followed and finally the pellet of RNA was dissolved in 20-100 µl of ddH_2_O. 

### Genotyping

The collection was screened using the specific primers. Primers for *met1* wild type were:

5΄-GCCTGGTCAAGTGGACTTCATC-3΄ and 5΄-CCATTCTTCACAGAGCATGCC-3΄, while they were 5΄-GATTGTGTCTCTACTACAGAGGC-3΄ and 5΄-TGGACGTGAATGTAGACACGTCG-3΄ for the mutant allele.

Polymerase chain reaction (PCR) reactions were performed in the volumes of 25 µl containing 10 ng DNA, 1X PCR buffer, 1.5 mM MgCl_2_, 0.2 mM dNTP, 0.4 µM each of primers forward and reverse and 0.625 U of DNA polymerase (Takara Shuzo Co., Japan). PCR program was performed as following: 94°C for 5 minutes, then 30 cycles of respectively 94°C for 30 seconds, 56°C for 30 seconds and 72°C for 30 seconds followed by 72°C for 10 minutes to complete DNA expansion. PCR products were visualized on 1% agarose gel. 

### Gene expression analysis

cDNA was synthesized using REVERTAID (Thermoscientific RevertAid cDNA synthesis Kit, USA) according to manufacturer instructions. Then, reverse transcription (RT)-PCR reactions were carried out using TaKaRa kit (TaKaRa, Japan) in 20 µl volume, according to manufacturer’s protocol. *actin* was used as an internal control in PCR reaction. Primers were: 

5΄-TGTGGATCTCCAAGGCCGAGTA-3΄ and 5΄-CCCCAGCTTTTTAAGCCTTTGATC-3΄ for *actin* and 5΄-CCTGTTTAGCCTGCTTTACA-3΄ and 5΄-GCAGAGAAAGGTCAATTTCA-3΄ for *TERT*. 

Quantitative RT-PCR (qRT-PCR) amplifications were carried out in the final volumes of 20 µl containing 10 ng cDNA, 0.4 µM of each primer and SYBR Green PCR Master Mix in iQ5 real thermocycler (Bio-Rad, USA). PCR condition was consisted of 94°C for 3 minutes, 50 cycles of 94°C for 10 seconds, 52°C for 10 seconds, 72°C for 10 seconds followed by 72°C for 10 minutes. 

### CpG island analysis

Selection of the *met1* gene was based on a bioinformatics survey. Thus, the -1000 to +100 region of *AtTERT* gene’s promoter was investigated using CpGPlot, CpGIF, PlantPan software for analysis of the methylation islands. The CpG islands found in the CpGPlot and CpGIF software were almost in line with each other. The CpNpG islands were not found in PlantPan software. Therefore, the met1 methyltransferase system, methylating CpG islands, was selected. 

### Statistical analysis

All experiments were repeated three times. Gene expression levels were analyzed using normalized calibrator method (21). Graphpad Prism version 6 (Graphpad Software, USA) was used for data analysis. A P value less than 0.05 was considered statistically significant. 

## Results

###  Wild type and mutant plants

**Fig.1:**
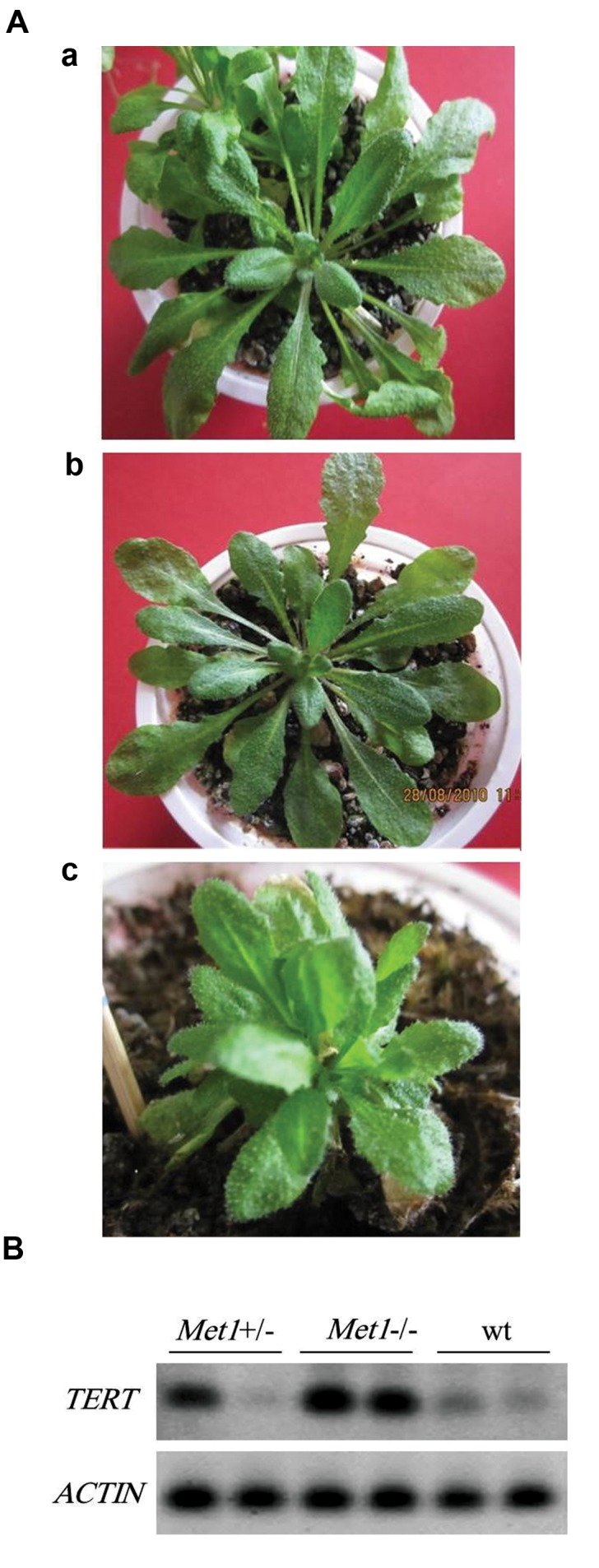
Morphology of wild-type, met1 homozygotes and heterozygotes. **A.** Wild type plant (a), heterozygous met1^+/-^ mutant plant (b), and homozygousmet1^-/-^ mutant plant (c). All of these plants are 60 days old seedlings and **B.** Reverse transcription polymerase chain reaction (RT-PCR) experiments of TERT transcript and *ACTIN* as an internal control (lower panel). The size of bands of *TERT* and *ACTIN* transcripts is 132 bp and 97 bp respectively.

After DNA extraction, genotyping was carried out. As expected amplified fragments size for wild type and mutant strains were 600 bp and 392 bp respectively. So, heterozygous and homozygous mutant plants were selected. Normal *met1* homozygous and heterozygous mutant plants are shown in Figure 1A. 

### TERT transcript level in the *met1* mutant plants

After RNA extraction, reverse transcription reaction was carried out. As we expected, gel electrophoresis showed respectively 97 bp and 132 bp bands regarding the *actin* and *TERT* genes (Fig .1B). 

For evaluating *AtTERT* gene, *actin* gene was used as a reference gene in this study. Cycle threshold (CT) value of reference gene was identical in wild type and mutant plants, indicating the equal expression of *actin* gene in the wild type and mutant plants. For TERT specific gene, different plants had different C_T_ values, indicating different gene expression in wild types, heterozygous and homozygous mutant plants. 

**Fig.2:**
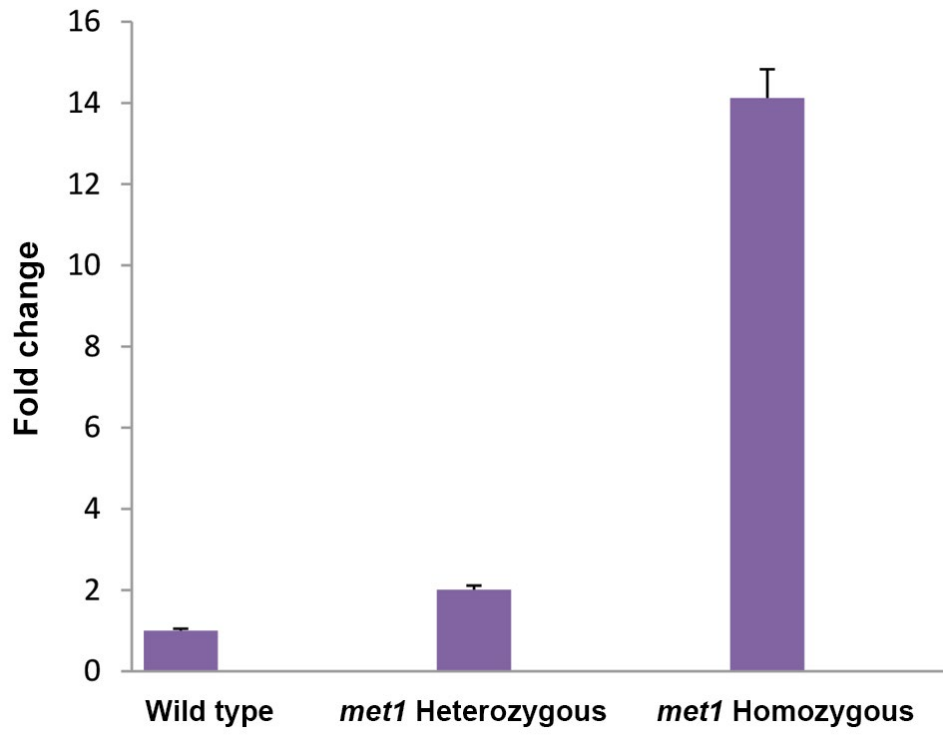
Telomerase gene expression patterns in the control, met1 heterozygous and homozygous mutant plants.

Telomerase gene expression levels in homozygous *met1* mutant plants showed 14.123 fold increase compared to the normal plants. Furthermore, *TERT* expression in heterozygous *met1* mutant plants was 2.009 fold higher than wild type plants (Fig .2).

## Discussion

DNA methylation is a major epigenetic mechanism and a key factor affecting normal development in animals and plants (22). Null mutations in the mouse *Dnmt1* or *Dnmt3a/b* genes lead to embryonic abortion but in *met1* mutant *Arabidopsis* plants are alive (23, 24). Therefore, *Arabidopsis* is an excellent genetic model for investigation of methylation defects. In the present study, we evaluated the effects of mutation of met1 DNA methyltransferase on telomerase gene expression. 

Telomerase promoter contains methylation islands within the region of -1000 and +100. Promoter hypermethylation mainly occurs in CpG sites and depends on met1 and drm2 methyltransferases (25). Therefore, the telomerase gene may be controlled by met1 system. 

Expression of TERT was increased ~14 fold in homozygous and ~2 fold in heterozygous met1 mutated plants. It seems that mutation in met1 methyltransferase systems decreases methylation of the promoter CpG islands of telomerase gene and increases the telomerase gene expression. Overexpression of telomerase leads to telomere lengthening. Long telomeres have detrimental effects on cells and special proteins, such as TZAP, triggered telomere trimming to a balanced level (26). The telomeric repeat sequences are bound to telomerebinding proteins; therefore, long telomere repeats would presumably recruit more of these proteins. Many telomerebinding proteins have non-relevant (non-telomeric) functions (27, 28). The increased telomere length leads to sequestration of telomere-binding proteins and inhibits their binding to non-telomeric sites. Indeed, long telomeres reduce growth and life span (29). Therefore, overexpression of telomerase in met1 mutated plants may be related to the limited growth and developmental abnormalities in Arabidopsis.

Further research is needed to show how homozygous mutant plants survive and grow, despite the severe developmental abnormalities. It is possible that other methylation systems compensate the effects of *met1* mutation and improves the phenotype. Hypermethylation, caused by *met1* mutation, inhibits the DNA demethylation pathway and activates the *de novo* methylation (12, 30, 31).

## Conclusion

 Our results suggest that TERT is a methyltransferaseregulated gene which may be involved in developmental abnormities caused by mutation in met1 methyltransferase system.
